# A case-control study on association of nucleotide excision repair polymorphisms and its interaction with environment factors with the susceptibility to non-melanoma skin cancer

**DOI:** 10.18632/oncotarget.20942

**Published:** 2017-09-15

**Authors:** Yan-Ling Li, Feng Wei, Yu-Ping Li, Li-Hua Zhang, Yan-Zhi Bai

**Affiliations:** ^1^ Department of Dermatology, The Second Hospital of Hebei Medical University, Shijiazhuang 050000, Hebei, People's Republic of China

**Keywords:** non-melanoma skin cancer, nucleotide excision repair, single nucleotide polymorphisms, interaction, smoking

## Abstract

**Aims:**

To investigate the association of several single nucleotide polymorphisms (SNPs) within nucleotide excision repair (*NER*) gene and additional gene- gene and gene- smoking interaction with non-melanoma skin cancer (NMSC) risk in a Chinese population.

**Methods:**

A total of 1322 participants (939 males, 383 females) were selected, including 660 NMSC patients and 662 control participants. Generalized multifactor dimensionality reduction (GMDR) was used to screen the best interaction combination among SNPs and smoking. Logistic regression was performed to investigate association between 4 SNPs within *NER* gene, additional gene- gene and gene- smoking interaction on NMSC risk.

**Results:**

NMSC risk was significantly higher in carriers with G allele of rs2228527 than those with AA genotype (AG + GG versus AA), adjusted OR (95%CI) =1.76 (1.24-2.37), and higher in carriers with the G allele of rs2228529 than those with AA genotype (AG + GG versus AA), adjusted OR (95%CI) = 1.66 (1.24-2.13). However, we did not find any direct association of the rs4134822 and rs1799793 with NMSC risk after covariates adjustment. GMDR model indicated a significant interaction combination (*p*=0.0010), including rs2228529 and current smoking. Overall, the cross-validation consistency of this model was 9/ 10, and the testing accuracy was 60.72%. Current smokers with rs2228529- GA or GG genotype have the highest NMSC risk, compared to never- smokers with rs2228529- AA genotype, OR (95%CI) = 2.92 (1.61-4.29).

**Conclusions:**

We found that the G allele of rs2228527 and the G allele of rs2228529 within *NER* gene, interaction between rs2228529 and current smoking were all associated with increased NMSC risk.

## INTRODUCTION

Non-melanoma skin cancer (NMSC), including squamous cell carcinoma (SCC) and basal cell carcinoma (BCC), ranks the most frequently diagnosed cancer worldwide, and the incidence rates of NMSC has become stabilized and was still increasing [1, 2]. Study has indicated that a personal history of NMSC is associated with increased risk of other malignancies [3], and NMSC may be a marker of a cancer-prone phenotype. Several biologic pathways may be disrupted by arsenic exposure, including interfering with the cell cycle activities of p53 or inhibiting base excision repair through reduced DNA ligase III or poly-(ADP-ribose) polymerase activity [4–6], the most compelling candidate for NMSC among Caucasians is the nucleotide excision repair (*NER*) pathway, given the specificity of *NER* to repair damage from exposure to ambient ultraviolet (UV) radiation. The importance of *NER* in the etiology of NMSC is well illustrated by xeroderma pigmentosum (XP), a disease in which rare, highly penetrant mutations disrupt the ability of *NER* to remove DNA photoproducts, leading to a 1,000-folds increased risk of NMSC [7, 8].

DNA repair systems, including *NER*, base excision repair, mismatch repair, and double-strand break repair, play a critical role in maintaining the stability and integrity of the genome [9, 10]. Previously, some studies [11–15] have reported the association between single nucleotide polymorphism (SNP) in *NER* genes and NMSC risk, but these studies concluded inconsistent findings. More than 80% of NMSC incidence could be attributed to ambient UV radiation [16], and several host features such as fair skin, red hair, melanocytic nevi, and family history have also been identified as risk factors [17, 18]. Cigarette smoking was considered as a risk factor for various chronic diseases, however, to date no study focused on the association of *NER* gene- smoking interaction with NMSC risk. So the aim of this study was to investigate the impact of several SNPs within *NER* gene, and their additional interaction with smoking on NMSC risk, based on a Chinese population.

## RESULTS

A total of 1322 participants (939 males, 383 females) were selected, including 660 NMSC patients and 662 control participants. The mean age of all participants was 63.9 ± 12.4 years. Table 1 shows the general and clinical characteristics for cases and controls participants. The means of age and BMI, distribution of males and current alcohol drinkers were not significantly different between cases and controls. The current smoking rate was higher in cases than controls.

**Table 1:** General characteristics for all study participants in NMSC cases and controls

Variables	NMSC cases (n=660)	Controls (n=662)	*P-values*
Age (years)	63.5±13.6	64.2±13.1	0.341
Males N (%)	464 (70.3)	475 (71.8)	0.561
BMI (kg/m^2^)	23.4±9.4	23.1±9.8	0.570
Current smokers, N (%)	255 (38.6)	190 (28.7)	0.0001
Current alcohol drinkers, N (%)	271 (41.1)	290 (43.8)	0.312
Basal cell carcinoma, N (%)			
1	440 (66.7)		
>1	220 (22.3)		
Squamous cell carcinoma, N (%)			
1	403 (61.1)		
>1	257 (38.9)		
SCC/BCC simultaneously, N (%)	356 (53.94)		

No significant difference in genotype frequencies from the Hardy–Weinberg equilibrium test was noted for any tested SNPs in the controls, all *p*- values were more than 0.05. The frequency for the G allele of rs2228527 was significantly higher in NMSC cases than controls (29.5% *vs*21.4%), and similarly, the frequency for the G allele of rs2228529 was also significantly higher in NMSC cases than controls (31.3% *vs*22.1%). Logistic regression analysis showed that NMSC risk was significantly higher in carriers with G allele of rs2228527 than those with AA genotype (AG + GG versus AA), adjusted OR (95%CI) =1.76 (1.24-2.37), and higher in carriers with the G allele of rs2228529 than those with AA genotype (AG + GG versus AA), adjusted OR (95%CI) = 1.66 (1.24-2.13). However, we did not find any direct association of the rs4134822 and rs1799793 with NMSC risk after covariates adjustment. (Table 2)

**Table 2:** Genotype and allele frequencies of 4 SNPs between case and control group

SNPs	Genotypes and alleles	Frequencies N (%)		OR (95%CI)*	*p- values*	*P-* values for HWE test in controls
		Controls (n=662)	Cases (n=660)			
rs2228527						
	Co-dominant					
	AA	410 (61.9)	332 (50.3)	1.00 (ref)		0.722
	AG	220 (33.2)	266 (40.3)	1.58 (1.19-2.04)	0.001	
	GG	32 (4.8)	62 (9.4)	2.01 (1.37-2.76)	<0.001	
	Dominant					
	AA	410 (61.9)	332 (50.3)	1.00 (ref)		
	AG+GG	252 (38.1)	328 (49.7)	1.76 (1.24-2.37)	<0.001	
	Allele, G (%)	284 (21.4)	390 (29.5)			
rs2228529						
	Co-dominant					
	AA	408 (61.6)	319 (48.2)	1.00 (ref)		0.138
	AG	215 (32.5)	272 (41.1)	1.52 (1.14-1.99)	0.0012	
	GG	39 (5.9)	71 (10.7)	1.91 (1.42-2.46)	<0.001	
	Dominant					
	AA	408 (61.6)	319 (48.2)	1.00 (ref)		
	AG+GG	254 (38.4)	343 (51.8)	1.66 (1.24-2.13)	<0.001	
	Allele, G (%)	293 (22.1)	414 (31.3)			
rs4134822						
	Co-dominant					
	GG	587 (88.7)	594 (89.7)	1.00 (ref)		0.122
	GA	75 (11.3)	68 (10.3)	1.12 (0.74-1.54)	0.624	
	AA	0 (0)	0 (0)			
	Allele, A (%)	75 (5.7)	68 (5.1)			
rs1799793						
	Co-dominant					
	AA	379 (57.2)	339 (51.4)	1.00 (ref)		0.127
	AG	234 (35.3)	255 (38.6)	1.20 (0.79-1.74)	0.579	
	GG	49 (7.4)	66 (10.0)	1.41 (0.82-2.06)	0.706	
	Dominant					
	AA	379 (57.2)	339 (51.4)	1.00 (ref)		
	AG+GG	283 (42.7)	321 (48.6)	1.22 (0.80-1.84)	0.628	
	Allele, G (%)	332 (25.1)	387 (29.3)			

* Adjusted for gender, age, BMI, smoking and alcohol drinking.

We also investigate the synergistic effect among 4 SNPs and current smoking using GMDR model. Table 3 indicated a significant two-locus model (p=0.0010) involving rs2228529 and current smoking, indicating a potential interaction between rs2228529 and current smoking on NMSC risk. Overall, the cross-validation consistency of this two- locus model was 9/10, and the testing accuracy was 60.72%. We also conducted stratified analysis for rs2228529 and current smoking using logistic regression. We found that current smokers with rs2228529- GA or GG genotype have the highest NMSC risk, compared to never- smokers with rs2228529- AA genotype, OR (95%CI) = 2.92 (1.61-4.29), after covariates adjustment (Table 4).

**Table 3:** GMDR analysis on the best gene- gene and gene–smoking interaction models

Locus no	Best combination	Cross-validation consistency	Testing accuracy	*p-values*
Gene- gene interactions^*^				
2	rs2228529 rs2228527	8/10	0.5399	0.0547
3	rs2228529 rs2228527 rs1799793	7/10	0.5399	0.1719
4	rs2228529 rs2228527 rs1799793 rs4134822	5/10	0.4958	0.3770
Gene- smoking interactions^*^				
2	rs2228529 current smoking	9/10	0.6072	0.0010
3	rs2228529 rs2228527 current smoking	7/10	0.4958	0.3770
4	rs2228529 rs2228527 rs1799793 current smoking	6/10	0.4958	0.4258
5	rs2228529 rs2228527 rs1799793 rs4134822 current smoking	5/10	0.5399	0.6230

*Adjusted for gender, age, BMI, smoking and alcohol drinking.

**Adjusted for gender, age, BMI and alcohol drinking.

**Table 4:** Stratified analysis for gene-smoking interaction by using logistic regression

rs2228529	Current smoking	OR (95% CI)^*^	*P-values*
AA	No	1.00	-
AG+GG	No	1.30 (1.04-1.72)	0.026
AA	Yes	1.45 (1.14-1.89)	<0.001
AG+GG	Yes	2.92 (1.61-4.29)	<0.001
Overall *p*- value for interaction= 0.0036			

*Adjusted for gender, age, BMI and alcohol drinking.

## DISCUSSION

In this study, we found that both the G allele of *ERCC*6- rs2228527 and the G allele of *ERCC*6- rs2228529were significantly associated with increased NMSC risk in a sample of the Chinese Han population. NMSC risks were significantly higher in carriers with G allele of *ERCC*6- rs2228527 than those with AA genotype, and higher in carriers with G allele of *ERCC*6- rs2228529 than those with AA genotype. However, we did not find any direct association of the rs4134822 and rs1799793 with NMSC risk after covariates adjustment. Previously, many studies have focused on the association between *NER* gene and some other diseases, including prostate cancer [19], gastric cancer [20] and thyroid cancer [21]. Several studies [11–15] were also performed on association between SNPs within *NER* gene and NMSC risks, but the limited number for these articles could not concluded a consistent result on this association, and could not clarify the mechanism between *NER* gene and NMSC susceptibility. Some studies support a non- significantly association between these variants and decreased risk of NMSC [14, 22], whereas others have found non- significant increases in NMSC risk [11, 23]. Previously, just one study [15] has reported evidence on the association between either rs2228527 or rs2228529 and NMSC, a thorough evaluation of *NER* gene variants revealed associations between two functional *ERCC*6 SNPs and NMSC, which to our knowledge have been reported firstly, and the increased risk was consistent among both men and women. The rs2228529 SNP falls within a highly conserved ubiquitin-binding domain in *ERCC*6 that is necessary for the transcription-coupled repair branch of *NER* [24]. a population-based study in New Hampshire with nearly 900 cases of BCC and 700 of SCC, observed by Miller et al [13] indicated a 15–25% decreased risk of either BCC or SCC among those with the *XPA* A23G (rs1800975) polymorphism. Another study also indicated that a 20% decreased risk of SCC and 10% decreased risk of BCC among those carrying variant forms of both *XPD* Lys751Gln and *Asp312Asn* [12], after adjusting for age, sex, skin pigmentation, and number of severe sunburns. Han et al [25] suggested a roughly 20% decreased risk for *XPD Asp312Asn* (rs1799793), whereas neither was associated with BCC.

NMSC susceptibility could be influenced by both genetic and environment factors, and previously several environmental factors associated with NMSC were reported, such as smoking, which was an important modifiable factor for NMSC [26, 27]. In current study, the smoking rate was higher in NMSC cases than controls, so we not only investigated gene- gene interaction on NMSC risk, but also gene- environment interaction between SNPs and smoking. We found a significant interaction between rs2228529 and current smoking on NMSC risk, current smokers with rs2228529- GA or GG genotype have the highest NMSC risk, compared to never- smokers with rs2228529- AA genotype. Smoking and *NER* SNPs appears to be the important risk factor for NMSC. Therefore, it is plausible that the coexistence of smoking and minor allele of rs2228529 contribute to the highest NMSC risk.

There several limitations in our study. Firstly, more participants should be included in this study, and the results obtained in this study should be checked by future studies with larger sample size. Secondly, some others environmental risk factors should be included in the gene- environment interaction detection, such as BMI, which was a new related factor for NMSC. Thirdly, sex differences on this association should be investigated in the future studies. Thirdly, the controls were selected from a population without any type of cancer who received physical examination in the hospital, but they would not be representative of the general population, so the selection bias may exist in this study. Lastly, the data on ultraviolet radiation (UVR) exposure was not collected in the investigation, so we could not exclude the influenced of UVR on the results.

In conclusion, we found that the G allele of rs2228527 and the G allele of rs2228529within *NER* gene, interaction between rs2228529 and current smoking were all associated with increased NMSC risk.

## MATERIALS AND METHODS

### Subjects

The study consisted of 1322 participants (939 males, 383 females), including 660 NMSC patients and 662 control participants. The mean age of all participants was 63.9 ± 12.4 years. Cases were defined as patients, aged 18 years and older, diagnosed with a histologically confirmed basal or squamous cell carcinoma between April 2010 and July 2015 and were recruited from the Second Hospital of Hebei Medical University. The controls were randomly selected from a population without any type of cancer, who received physical examination in our hospital and 1:1 matched to cases on the basis of age (±3 years) and sex. Individuals recruited to the control group had no previous history or family history of cancer. Both the NMSC cases and controls were unrelated Han Chinese population. Questionnaire investigation was conducted for all participants, and data on demographic information, clinical and biochemical index and life style for all participants were obtained. Body weight, height were measured. Blood samples were collected from each participant in the morning after at least 8 hours of fasting. Informed consent was obtained from all participants.

### Genomic DNA extraction and genotyping

The NCBI database (http://www.ncbi.nlm.nih.gov/projects/SNP) is used for SNPs selection according to the following methods: 1) located in a gene fragment that could have functional effects; 2) MAF more than 5%; 3) previously reported associations with NMSC, but is not well studied. In this study, four SNPs within *NER* gene were selected for genotyping, including: *ERCC*6- rs2228529 (*Arg1213Gly*), *ERCC*6- rs2228527 (*Gln1413Arg*), *XPD*- rs1799793 (*Asp312Asn*) and *XAB2/XPA*- rs4134822 (*Val126Ile*). Genomic DNA from participants was extracted from EDTA-treated whole blood, using the DNA Blood Mini Kit (Qiagen, Hilden, Germany) according to the manufacturer's instructions. Genotyping for rs2228529, rs2228527 and rs4134822 were detected by Taqman fluorescence probe. Probe sequences of all SNPs were shown in Table 5. ABI Prism7000 software and allelic discrimination procedure was used for genotyping of fore-mentioned three SNPs. Probes for the 3 SNPs were listed in Table 5. A 25μl reaction mixture including 1.50ul SNP Genotyping Assays (20×), 12.25μl Genotyping Master Mix (2×), 20ng DNA, and the conditions were as follows: initial denaturation for 10 min and 93°C, denaturation for 15 s and 90°C, annealing and extension for 120 s and 65°C, 60 cycles. Genotyping for rs1799793 was performed by polymerase chain reaction (PCR) restriction fragment length polymorphism (PCR-RELP) method. The primers and PCR conditions are shown in Table 1. The PCR conditions were as follows: 95°C for 5 min; 30 cycles of 95°C for 30s, 60°C for 30s, and 72°C for 30s; and a final extension step of 72°C for 10 min. Genotyping results were confirmed by randomly assaying 10% of the original specimens for replication to exclude genotyping errors. There were no discrepancies between genotypes determined in duplicate.

**Table 5:** Probe and primer sequences for 4 SNPs used for genotyping

SNP ID	Gene	Chromosome	Functional consequence	Primer/probe sequences
rs2228527	*ERCC6* *Arg1213Gly*	10:49470323	Missense	5′-AAAGCCTAAGAACTCTAAGCATTGC[A/G] GAGACGCCAAGTTTGAAGGAACTCG-3′
rs2228529	*ERCC6* *Gln1413Arg*	10:49459059	Missense	5′- TTAGAAAGTGAAAGCGGGCACCTGC[A/G] GGAAGCTTCTGCCCTGCTGCCCACC-3′
rs4134822	*XAB2/XPA* *Val126Ile*	19:7627389	Missense	5′- CCAGTTCCTCATGGACCAGGGGCGC[A/G] TCACACACACCCGCCGCACCTTCGA-3′
rs1799793	*XPD* *Asp312Asn*	19:45364001	Missense, nc transcript variant	F: 5′-CAGCTCATCTCTCCGCAGGATCAA-3′ R: 5′-GTCGGGGCTCACCCTGCAGCACTTCCT-3′

### Statistical analysis

Categorical variables were presented as absolute values and percentages, and continuous variables were expressed as means ± standard deviations (SD). Student's t test was used to compare continuous variables, while Chi-square test was used to compare categorical variables between cases and controls. Hardy-Weinberg equilibrium (HWE) examination was used by SNPstats (http://bioinfo.iconcologia.net/SNPstats). Generalized multifactor dimensionality reduction (GMDR) was used to screen the best interaction combination among SNPs and current smoking. Logistic regression was performed to investigate the impact of 4 SNPs within *NER* gene on NMSC risk, and additional stratified analysis for gene- current smoking interaction on NMSC risk. Two sided test with *P* < 0.05 was considered statistically significant.
